# Association between atherosclerotic cardiovascular disease score and skin carotenoid levels estimated via refraction spectroscopy in the Japanese population: a cross-sectional study

**DOI:** 10.1038/s41598-024-62772-y

**Published:** 2024-05-28

**Authors:** Akira Obana, Mieko Nakamura, Ayako Miura, Miho Nozue, Shigeki Muto, Ryo Asaoka

**Affiliations:** 1 Department of Ophthalmology, Seirei Hamamatsu General Hospital, 2-12-12 Sumiyoshi, Chuo-ku, Hamamatsu City, Shizuoka 430-8558 Japan; 2 Department of Medical Spectroscopy, Institute for Medical Photonics Research, Preeminent Medical Photonics Education and Research Center, Hamamatsu University School of Medicine, 1-20-1 Handayama, Higashi-ku, Hamamatsu City, Shizuoka 431-3192 Japan; 3 Department of Community Health and Preventive Medicine, Hamamatsu University School of Medicine, 1-20-1 Handayama, Higashi-ku, Hamamatsu City, Shizuoka 431-3192 Japan; 4 Faculty of Health Promotion Sciences, Department of Health and Nutritional Sciences, Tokoha University, 1230 Miyakoda-cho, Hamana-ku, Hamamatsu City, Shizuoka 431-2102 Japan; 5 Seirei Center for Health Promotion and Prevention Medicine, Seirei Social Welfare Community, 2-35-8 Sumiyoshi, Chuo-ku, Hamamatsu City, Shizuoka 430-0906 Japan

**Keywords:** Health examination participants, Cardiovascular disease risk, Skin carotenoids, Veggie meter, Logistic regression analysis, Japanese, Cardiology, Health care

## Abstract

Carotenoids play a role in preventing and impeding the progression of atherosclerotic cardiovascular diseases (ASCVDs) through their anti-oxidative effects. This study evaluated associations between ASCVD risk and skin carotenoid (SC) levels, reflecting dietary carotenoid intake. Participants’ ASCVD risk was assessed using the Hisayama ASCVD risk prediction model, and SC levels were measured through a reflection spectroscope (Veggie Meter). The associations between high ASCVD risk and SC levels were analyzed using logistic regression analysis and a restricted cubic spline (RCS) model. A total of 1130 men and women (mean age: 56 years) from participants who underwent a health examination in Seirei Center for Health Promotion and Prevention Medicine in 2019 and 2022 were analyzed. Of these, 4.6% had moderate or high ASCVD risk. Mean SC values were 236, 315, 376, 447, and 606 in quintile Q1 to Q5, respectively. The adjusted odds ratios (95% confidence intervals) of SC quintile for moderate- or high-risk ASCVD was 0.24 (0.12–0.51) in Q5 (495 ≤), 0.42 (0.23–0.77) in Q4, 0.50 (0.29–0.88) in Q3, and 0.68 (0.41–1.12) in Q2 compared to Q1 (< 281). High SC values continuously showed non-linear inverse association with moderate- or high-risk for ASCVD in Japanese adults. Non-invasive SC measurements may be a good indicator for recommending carotenoids to prevent cardiovascular disease.

## Introduction

Carotenoids are phytonutrients comprising eight isoprene molecules and contain 40 carbon atoms. Their 9–11 conjugated double bonds provide an anti-oxidative function by quenching singlet oxygen and scavenging free radicals^1,2^. Carotenoids also possess anti-inflammatory effects in organisms^3,4^. Among more than 750 carotenoids, approximately 40 types are detected in the human body, and several studies elucidated the association between blood carotenoid concentrations and numerous diseases and mortality^5–8^. Bohn et al. reported that a serum carotenoid concentration of > 1725 nmol/L was required for maintaining health^9^, and Donaldson reported that a serum carotenoid concentration of > 2500 nmol/L decreases the risk of cardiovascular disease (CVD), cancer, and other chronic diseases whereas a concentration of < 1000 nmol/L increases the risk of the diseases mentioned above^10^.

Atherosclerotic cardiovascular diseases (ASCVD) are disorders caused by cholesterol plaque formation in the arterial walls and its disruption by thrombosis. ASCVD is a single pathologic entity that affects different vasculatures in the body, which encompasses coronary heart disease and atherothrombotic brain infarction^11^. Atherosclerosis is induced in part by low-density lipoprotein oxidation^12^, and oxidative stress considerably contributes to atherosclerosis and CVD onset^13^. Since high serum carotenoid concentrations and their antioxidant capacity have been reported to suppress early-stage atherosclerosis^14^ and CVD^15^, carotenoids play a role in the prevention and impeding the progression of ASCVD^16,17^.

In previous studies, carotenoids in the body were quantified by blood (serum or plasma) carotenoid concentrations. Although blood carotenoid concentrations can be accurately measured by high performance liquid chromatography, the measurement is costly, and taking blood samples is invasive. Conversely, skin carotenoid (SC) level measurement is known to be a non-invasive rapid screening alternative that can be used in large-scale studies and dietary education. SC measurements can be conducted using resonance Raman spectroscopy (RRS) or reflection spectroscopy (RS) methods^18–23^. SC levels measured using RRS and RS are strongly correlated with blood carotenoid concentrations^24^. RRS requires laser excitation and highly sensitive detection schemes to measure the Raman signals of carotenoid vibration which are highly expensive. Conversely, a pressure-mediated RS can measure SC levels with less complexity and cost than RRS. Therefore, RS is advantageous for its use in public health studies and studies with preschool and school children^25–29^.

The association between SC and CVD risk was observed in Singapore^30^, and we reported the association between SC measured using RS and metabolic syndrome in the Japanese population during a health examination previously^31^; in this study, the dose–response association between ASCVD risk and SC levels in the same population of participants were analyzed. The results obtained in this study enhance the relevance of SC measurements, providing valuable data for using SC measurements in nutrition education aimed at improving vegetable intake and health promotion.

## Methods

### Participants

**Figure 1:**
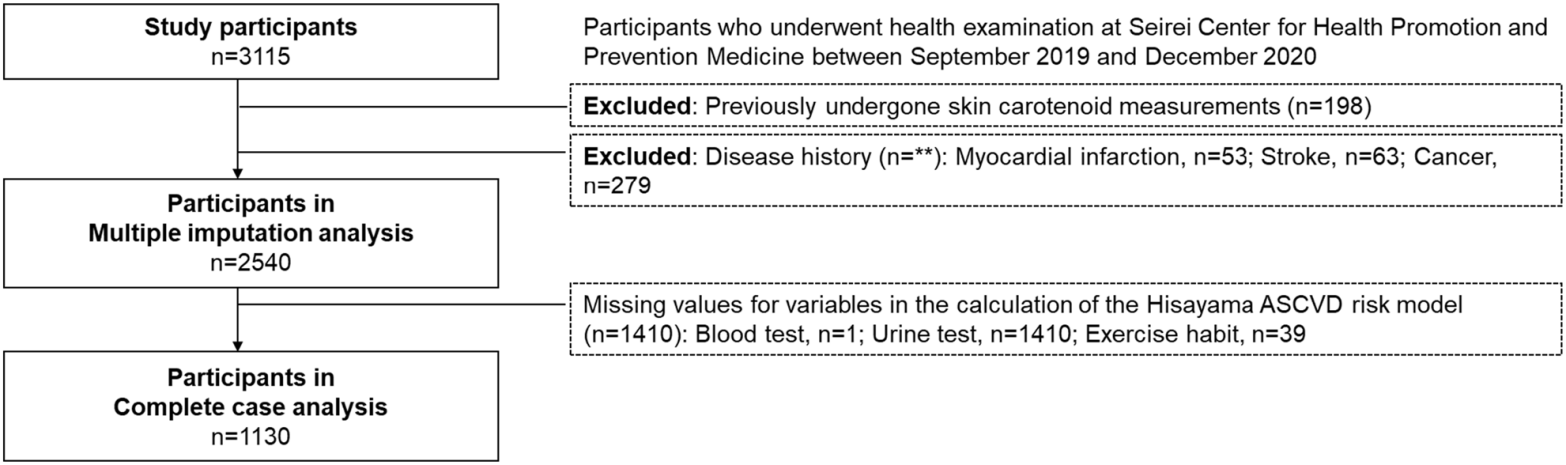
Flowchart of the participants.

Among the 3115 participants who underwent a health examination in Seirei Center for Health Promotion and Prevention Medicine from September 2019 to December 2020, 2540 participants who underwent SC measurement for the first time and without a history of myocardial infarction (n = 53), stroke (n = 63), and cancer (n = 279) were included in the study. A participant flowchart is shown in Fig. 1. Some of the participants were the same respondents investigated in our previous study^31^. The participants were interviewed by trained medical staff regarding their lifestyle, disease history, use of antihypertensive agents, antihyperlipidemic agents, oral diabetes drugs, and insulin, smoking status, alcohol drinking status, and regular exercise. The participants underwent physiological examinations, including body mass index (BMI), systolic and diastolic blood pressure, SC level measurement, and blood tests.

The current cross-sectional study complied with the tenets of the Declaration of Helsinki. The Institutional Review Boards of Seirei Hamamatsu General Hospital and Seirei Center for Health Promotion and Prevention Medicine approved the research (IRB No. 3030, 31–02). All participants provided written informed consent for inclusion in the study.

### ASCVD risk assessment

ASCVD risk was assessed using the Hisayama ASCVD risk prediction model^32^, which was adopted from the Japanese Arteriosclerosis Society Guidelines for the Prevention of Arteriosclerotic Cardiovascular Disease 2022 (https://www.j-athero.org/jp/jas_gl2022/). In this model, a 10-year probability of developing ASCVD events was calculated in each participant using a multivariable formula with age (in years), sex, systolic blood pressure (in mmHg), diabetes, serum high-density lipoprotein (HDL) cholesterol (in mg/dL), serum low-density lipoprotein (LDL) cholesterol (in mg/dL), protein urea, current smoking, and regular exercise habit. Additionally, a Hisayama ASCVD risk score was calculated by simply adding the scores corresponding to the categories of each of the above variables except age, that is, sex (women: 0; men: 7 points), systolic blood pressure (< 120 mmHg: 0; 120–129 mmHg: 1; 130–139 mmHg: 2; 140–159 mmHg: 3; 160 mmHg: 4 points), diabetes (no: 0; yes: 3 points), serum HDL cholesterol (60 mg/dL: 0; 40–59 mg/dL: 1; < 40 mg/dL: 2 points), serum LDL cholesterol (< 120 mg/dL: 0; 120–139 mg/dL: 1; 140–159 mg/dL: 2; 160 mg/dL: 3 points), proteinuria (no: 0; yes: 4 points), current smoker (no: 0; yes: 2 points), and regular exercise (yes: 0; no: 2 points)^32^. The classification was based on the presence or absence of proteinuria and diabetes, the distinction of smoking habit into those who smoke one or more cigarettes per day or those who do not, and the categorization of regular exercise to individuals engaging in sports or other forms of activity at least three times a week or not^32^. However, in our study, regular exercise was defined as light exercise of 30 min or more, performed at least twice a week for at least 1 year. In the Hisayama ASCVD risk prediction model, a 10-year probability of developing an ASCVD event is defined as “moderate risk” if the probability is more than 2% and “high risk” if the probability is more than 10%, considering the incidence of ASCVD in the Japanese population. In this study, “moderate or high risk” was defined as a Hisayama ASCVD risk score of 15 or higher, with a 10-year probability of developing an ASCVD event of at least 2% among participants aged 40–49 years.

When calculating the Hisayama score and the ASCVD probability, there were missing values for blood tests (n = 1), urine tests (n = 1410), and exercise habits (n = 39), resulting in a total of 1130 participants for complete case analysis without missing values. Additionally, owing to the large number of missing values, a multiple imputation analysis was also conducted, with the variables used for the Hisayama score and probability calculations to complement the missing values (20 sets were created; n = 2540).

### SC level measurement

The SC levels were measured using Veggie Meter (VM) (version 2.0., serial number, 618W0091, Longevity Link Corporation, Salt Lake City, Utah) as VM scores (range: 0–1200, arbitrary unit), according to the manufacturer’s instructions. Calibration was conducted with the manufacturer-provided reference materials before daily measurements twice daily (before the morning and afternoon sessions)^33^. All participants washed their hands with soap and disinfected their fingers with a disinfectant. The researcher then assessed the hands of the participants, ascertaining the absence of contamination. The participants then inserted their left middle finger into the device’s finger cradle and had the tip pushed against the convex contact lens surface with the aid of a spring-loaded lid. The modest pressure applied to the fingertip reduced the blood perfusion of the measured tissue volume, preventing the strongly absorbed blood from interfering with SC level measurements. The VM scores were determined as the average of three consecutive measurements. The obtained VM scores were converted to values with our standard VM (version 1.0., serial number, 415W0156-1) using the regression equation of our previous study^34^, and compensated values were used for analyses.

### Statistical analysis

Participant characteristics by SC quintiles were examined using the Chi-square test for categorical variables and analysis of covariance for continuous variables. The association between moderate or high risk for ASCVD (15 or more of the Hisayama ASCVD risk score) and SC quintiles (Q1 to Q5, Q1: reference) was estimated using logistic regression analysis adjusted for age and BMI, which were excluded from the calculation of the Hisayama ASCVD risk score. Trend-p values were obtained with SC quintiles as continuous variables. Additionally, the non-linear association between moderate or high risk for ASCVD and SC levels (arbitrary unit) was estimated using logistic regression analysis adjusting for age and BMI in a restricted cubic spline (RCS) model with three knots (0.10. 0.50, 0.90) in complete case analysis.

Data were analyzed using IBM SPSS Statistics for Windows, version 28.0 (IBM Corp., Armonk, NY, USA) and R software, version 4.1.3. All tests were two-tailed, and statistical significance was set at P < 0.05.

## Results

A total of 499 men and 631 women were analyzed in a complete case analysis. The mean (standard deviation, SD) of age was 56.4 (11.0) years (men: 57.0 [10.2]; women: 56.0 [11.5]), and the mean (SD) of the SC values expressed as VM score was 395.5 (135.0) (men: 371.1 [129.5]; women 414.7 [136.2]). The characteristics of the participants according to SC quintile are shown in Table 1. The mean SC values (range) were 236 (< 282), 315 (282 ≤ and < 345), 376 (345 ≤ and < 407), 447 (407 ≤ and < 495), and 606 (495 ≤) in quintiles Q1 to Q5, respectively. Male sex, young age, and current smokers were more likely to belong in the lower quintile of the SC, whereas people who do regular exercise were less likely to belong in the lower quintile of the SC. Diastolic blood pressure, serum triglycerides, and BMI were higher, while serum HDL cholesterol was lower in the lower quintile of the SC.

**Table 1:** Characteristics of the participants according to skin carotenoid quintile.

	Skin carotenoid quintile										
	Q1		Q2		Q3		Q4		Q5		p-value
N	229		228		221		226		226		
Skin carotenoid value, range	< 282		282 ≤ & < 345		345 ≤ & < 407		407 ≤ & < 495		495 ≤		
Skin carotenoid value, mean, SD	236.4	37.2	315.0	19.2	375.6	17.1	446.7	25.6	605.9	94.4	< 0.01
Sex, men, N, %	130	56.8	114	50.0	96	43.4	88	38.9	71	31.4	< 0.01
Age, years, mean, SD	53.6	9.7	53.9	10.3	57.4	10.0	57.1	11.8	60.3	11.5	< 0.01
Systolic blood pressure, mmHg, mean, SD	118.1	16.0	116.7	15.2	117.3	16.3	116.6	15.1	116.8	14.8	0.81
Diastolic blood pressure, mmHg, mean, SD	73.8	10.7	72.7	11.1	72.8	10.7	71.6	9.1	70.9	9.5	0.03
Use of antihypertensive drugs, n, %	34	14.8	31	13.6	34	15.4	37	16.4	43	19.0	0.59
Diabetes, n, %	16	7.0	6	2.6	11	5.0	7	3.1	7	3.1	0.11
Serum total cholesterol, mg/dL, mean, SD	216.1	35.7	216.0	30.3	213.7	34.8	216.0	34.2	219.3	33.4	0.53
Serum LDL cholesterol, mg/dL, mean, SD	128.5	32.4	129.6	28.8	126.1	29.5	128.0	29.3	128.1	29.4	0.81
Serum HDL cholesterol, mg/dL, mean, SD	69.2	20.2	71.6	18.6	72.2	19.4	74.0	18.6	77.3	19.3	< 0.01
Serum triglycerides, mg/dL, mean, SD	116.7	142.5	95.4	56.9	95.4	54.0	87.1	44.4	86.2	45.4	< 0.01
Body mass index, kg/m^2^, mean, SD	23.4	3.5	22.9	3.4	22.7	3.4	21.9	3.0	21.4	3.0	< 0.01
Proteinuria, n, %	15	6.6	13	5.7	7	3.2	9	4.0	8	3.5	0.35
Current smoker, n, %	24	10.5	8	3.5	9	4.1	1	0.4	0	0.0	< 0.01
Regular exercise, n, %	68	29.7	73	32.0	81	36.7	81	35.8	104	46.0	< 0.01

Skin carotenoid was estimated via refraction spectroscopy, Veggie Meter.

Chi-square test was used for the categorical variables and analysis of covariance was used for the continuous variables.

*SD* standard deviation, *LDL* low-density lipoprotein, *HDL* high-density lipoprotein.

**Figure 2:**
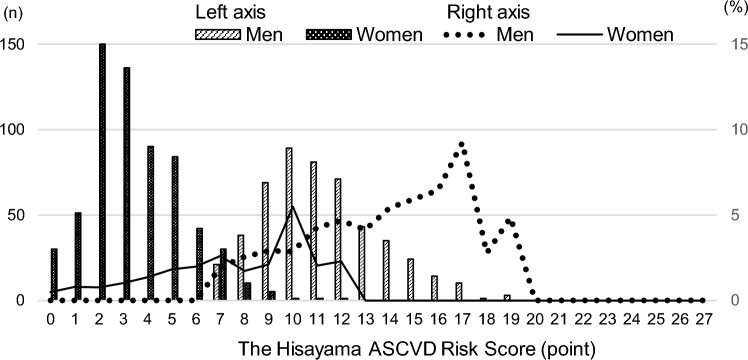
Distribution of Hisayama ASCVD Risk Score and mean 10-year probability of developing an ASCVD event using the score. Left axis is the frequency of the Hisayama ASCVD Risk Score. Right axis is the mean 10-year probability of developing an ASCVD event obtained using multivariable formula of Hisayama ASCVD Risk Prediction Model^32^.

The distribution of the Hisayama ASCVD Risk Score and the mean 10-year probability of developing an ASCVD event using the score for the complete case are shown in Fig. 2. 4.6% of participants had a Hisayama ASCVD Risk Score of 15 or more and were identified as moderate or high risk for ASCVD.

**Figure 3:**
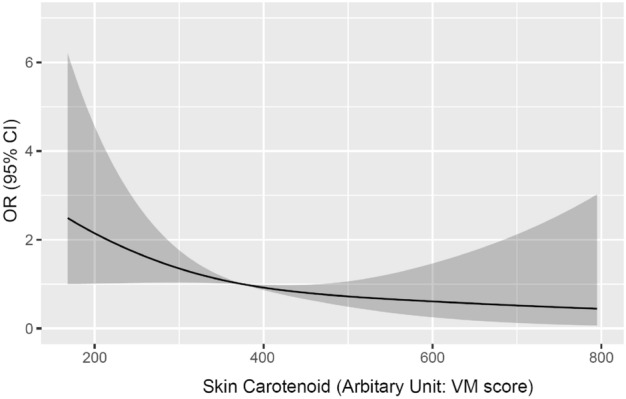
Association between moderate or high risk for ASCVD and skin carotenoid values. *OR* odds ratio, *CI* confidence interval. Skin carotenoid was estimated via refraction spectroscopy, Veggie Meter. Moderate or high risk for ASCVD was defined as 15 or more points on the Hisayama ASCVD Risk Score^32^.

The association between moderate or high risk for ASCVD and SC quintile using logistic regression analysis is shown in Table 2. The adjusted odds ratios of SC for moderate- or high-risk ASCVD was 0.25 (95% confidence interval [CI], 0.08–0.77) in Q5, 0.42 (95% CI, 0.17–1.03) in Q4, 0.46 (95% CI, 0.20–1.06) in Q3, and 0.46 (95% CI, 0.21–1.02) in Q2 compared to Q1 as reference in the complete case analysis. The results were almost similar in the multiple imputation analysis; however, the association was significant in Q3 and Q4 in addition in Q5. The RCS model demonstrated a non-linear inverse dose–response relationship between moderate- or high-risk ASCVD and SC values after adjusting for age and BMI (Fig. 3).

**Table 2:** Odds ratios and 95% confidence intervals for moderate- or high-risk ASCVD with skin carotenoid quintile.

Skin carotenoid quintile	Crude				Age- and body mass index-adjusted			
	OR	95% CI		Trend	OR	95% CI		Trend
		LL	UL	p-value		LL	UL	p-value
Complete case analysis								
Q1	1			< 0.01	1			< 0.01
Q2	0.43	0.20	0.93		0.46	0.21	1.02	
Q3	0.40	0.18	0.89		0.46	0.20	1.06	
Q4	0.30	0.13	0.72		0.42	0.17	1.03	
Q5	0.17	0.06	0.50		0.25	0.08	0.77	
Multiple imputation analysis								
Q1	1			< 0.01	1			< 0.01
Q2	0.58	0.34	0.99		0.63	0.36	1.08	
Q3	0.46	0.24	0.90		0.50	0.25	0.99	
Q4	0.38	0.19	0.74		0.48	0.24	0.96	
Q5	0.22	0.08	0.60		0.28	0.10	0.82	

Skin carotenoid was estimated via refraction spectroscopy, Veggie Meter.

Moderate or high risk for ASCVD was defined as 15 or more points on the Hisayama ASCVD Risk Score^32^.

## Discussion

In this cross-sectional study, SC was estimated using a commercially available RS (VM), and ASCVD risk was evaluated using the Hisayama ASCVD risk prediction model. We found that the mean SC values (VM scores) were 395.5, and 4.6% of the participants had moderate- or high-ASCVD risk. The OR for moderate- or high-ASCVD risk was statistically low in the highest quintile of SC compared to the lowest quintile in the complete case analysis. Additionally, the RCS model showed a non-linear inverse dose–response relationship between the moderate- or high-risk ASCVD and SC values, and the ASCVD risk increased more rapidly, when the SC value was low.

The inverse association between SC and CVD risk observed in this study corroborates with the results of a study conducted in Singapore by Toh et al.^30^ despite differences in SC measurement and CVD risk assessment methods. That is, they measured SC using RRS, and we measured it using the simpler RS (VM), and they evaluated CVD risk using the Framingham Risk Score, and we assessed it using the Hisayama ASCVD risk score developed for the Japanese population. The inverse association between SC and CVD risk, commonly observed regardless of the measurement or assessment method, infers that the association is robust. East Asian countries, such as Japan, Korea, and China, have lower coronary heart disease (CHD) mortality rates than Western countries, including the United States and the United Kingdom; in contrast, stroke is generally more common in East Asian countries than in Western countries^35^. Major risk factors for CVD, such as hypertension, hypercholesterolemia, diabetes, and smoking, are common in both Western and East Asian countries. Many models for predicting CVD risk, such as the Framingham Risk Score, have been developed worldwide^36^, and numerous risk prediction models have also been developed in Japan^32,37–41^. Of these, the Hisayama ASCVD risk prediction model^32^ was adopted in this study because it predicts atherothrombotic brain infarction in addition to CHD and provides both a 10-year probability of developing ASCVD events using a multivariable formula and a simple risk score that can be evaluated by age group.

As shown in Fig. 3, the risk for ASCVD decreases with higher SC levels; SC values in the highest quintile, specifically a VM score of 495 or higher, were potentially effective in CVD prevention. Evidence on the benefits of SC in preventing CVD or maintaining health is currently lacking. However, several reports examined the association between blood carotenoid concentrations and CVD and other diseases^5–10,42–45^. To estimate the VM score from plasma carotenoid concentrations, Rush et al.^46^ created a regression equation. According to this regression equation, the serum carotenoid level of 2500 nmol/L, which was reported as effective in preventing CVD and other diseases by Donaldson^10^, equivalent to 310. Rush et al. proposed VM scores of 530 which were desirable for 2011 #196}, might be equivalent to 451, and 1725 nmol/L, which was reported effective for health maintenance by Bohn et al.^9^, might be health maintenance^46^. In this study, the result of a favorable VM score of 495 or higher is broadly consistent with the favorable VM score predicted by the estimation equation from serum carotenoids. The validity of this value needs to be verified in the future by other populations and by cohort and intervention studies.

VM has been widely used in the field of nutrition because VM can measure SC levels non-invasively in a short time. Several studies^27,47–51^ clarified the association between fruit and vegetable intake and SC levels measured by VM and some interventional studies^52,53^ revealed that VM accurately detects differences in carotenoid intake through diet. In our previous study^54^, informing participants of their VM scores has proven effective in motivating fruit and vegetable intake, and repeated measurements have increased SC levels. In addition to these nutritional approaches, SC level identification as an effective measure for preventing various diseases would serve as a valuable indicator in nutritional guidance towards a diet beneficial for disease prevention.

The major limitation of this study was the cross-sectional observational nature of the study; thus, we cannot infer the causal relationship between SC and ASCVD risk. Furthermore, the study population may be more health-conscious, as they were selected from those who had undergone a health check-up. Since the variables used to calculate the Hisayama ASCVD risk score and probability had many missing values, we added a multiple imputation analysis. The results demonstrated a more evident association between SC and ASCVD risk.

In conclusion, high SC levels, estimated by VM, continuously showed a non-linear inverse association with moderate- or high-risk ASCVD in Japanese adults. The highest quintile of SC (495 ≤ in VM score) was associated with low risk of ASCVD and the ASCVD risk increased more rapidly, when the SC value was low. Whether CVD occurrence can be predicted from skin carotenoids requires further research. However, given the numerous reports on the CVD preventive effects of fruit and vegetable consumption, non-invasive measurement of SC may be an optimal indicator for recommending adequate fruit and vegetable intake for CVD prevention.

### Supplementary Information

Supplementary Information.

## Data Availability

Data described in the manuscript were substituted as supplement data.
